# Interaction rules underlying group decisions in homing pigeons

**DOI:** 10.1098/rsif.2013.0529

**Published:** 2013-12-06

**Authors:** Benjamin Pettit, Andrea Perna, Dora Biro, David J. T. Sumpter

**Affiliations:** 1 Department of Zoology, University of Oxford, Oxford, UK; 2 Department of Mathematics, Uppsala University, Uppsala, Sweden

**Keywords:** collective animal behaviour, leadership, bird flocks, collective decision-making, self-propelled particles

## Abstract

Travelling in groups gives animals opportunities to share route information by following cues from each other's movement. The outcome of group navigation will depend on how individuals respond to each other within a flock, school, swarm or herd. Despite the abundance of modelling studies, only recently have researchers developed techniques to determine the interaction rules among real animals. Here, we use high-resolution GPS (global positioning system) tracking to study these interactions in pairs of pigeons flying home from a familiar site. Momentary changes in velocity indicate alignment with the neighbour's direction, as well as attraction or avoidance depending on distance. Responses were stronger when the neighbour was in front. From the flocking behaviour, we develop a model to predict features of group navigation. Specifically, we show that the interactions between pigeons stabilize a side-by-side configuration, promoting bidirectional information transfer and reducing the risk of separation. However, if one bird gets in front it will lead directional choices. Our model further predicts, and observations confirm, that a faster bird (as measured from solo flights) will fly slightly in front and thus dominate the choice of homing route. Our results explain how group decisions emerge from individual differences in homing flight behaviour.

## 1. Introduction

Bird flocking is an intriguing and spectacular collective phenomenon. Some bird species, such as starlings, can form large groups that move together in a coordinated way, with changes of direction propagating quickly through the entire flock [1]. Many species flock together during migration and remain cohesive over long distances. In order to stay together in a coordinated flock, individual birds need to respond appropriately to their neighbours' positions and directions of movement. Modelling studies have investigated flocking interactions through a class of models known as self-propelled particle (SPP) models [2–7]. These models, largely inspired by statistical physics, simulate individuals as particles that interact locally with their neighbours. Usually, a simple set of interaction rules is sufficient to reproduce realistic collective patterns [3,7], including cohesive flocks in which information propagates through the entire group [4].

From a functional perspective, animals moving in groups benefit in several different ways from staying together and moving cohesively. These include an increased ability to detect and avoid predators [8] or to reach a target destination [4,9]. However, in order to stay with the others, individuals have to balance their own preferences against the benefit of staying in a group, for instance, when negotiating a common direction of movement or a common activity [10]. There is an extensive biological literature on how such consensus decisions are achieved. The focus has been either on the mechanisms involved in reaching consensus (including nonlinear, quorum-sensing type responses [11]), or on individual differences that affect an animal's weight in a group decision [12–15]. Although many features of collective decision-making do not require heterogeneity in individual behaviour, consistent individual differences in leadership have been found in a range of species, including pigeons [16,17], mosquitofish [18], zebras [12] and several species of primates [13].

By leadership, we mean that some individuals have more influence over a group decision, inferred from the fact that the group's choice reflects those individuals' information or preferences. This definition of leadership does not imply any particular mechanism. Importantly, a group decision can display leadership without the group members actively choosing a leader. Simulations by Conradt *et al.* [5] demonstrate several types of heterogeneity that cause self-organized leadership, without the need for global communication or individual recognition. These fall into the categories of ‘leading by need’ (stronger attraction to a target stimulus) and ‘leading by social indifference’ (weaker response to conspecifics). The two categories are based on contrasting functional priorities of the individual: the importance allocated to reaching the target versus the importance of remaining with the rest of the group.

We cannot understand group decision-making without understanding the underlying interactions among individuals. The interaction rules that a particular species has evolved will reflect a trade-off between various features of collective behaviour, such as group cohesion, the speed and accuracy of group decisions, and an individual's ability to seek cover from predators within the ‘selfish herd’ [8,19,20]. Because of these competing selection pressures, it is not clear that interaction rules will always optimize collective information processing. Nonetheless, making mechanistic links between measured interaction rules and group outcomes will help us discover the functional significance of the interaction rules, for example, whether they optimize tracking a gradient [21] or avoiding predation [20]. If we can explain the positioning of individuals within the group in terms of their interactions, then we may also be able to make a link between interaction rules and information processing at the group-level. To illustrate this, let us consider a group of only two individuals. Assuming that there is a blind visual angle, information transfer will be unidirectional if they travel one behind the other, whereas moving side by side they can see each other, which enables bidirectional information transfer.

Homing pigeons provide an excellent system for testing how movement interactions determine group decisions. They can be tracked with high spatial and temporal accuracy under field conditions, and their route-learning behaviour can be used to set up group decision-making experiments over a scale of kilometres [17]. When a pigeon is released far from its home loft, it heads back home, relying on a variety of different sensory cues [22]. If a pigeon is released many times from the same site, it usually learns a stereotyped route back to the loft, with varying degrees of similarity among the routes of different birds (as in [23]). This creates a conflict of information when a pair of pigeons flies home together. Biro *et al.* [17] found that pairs take a compromise route if their previous solo routes are close together, but above a critical distance one bird leads the other. Further studies found that navigational certainty and experience with the local landscape give a pigeon more influence over a pair's choice of homing route [14,15]. Tracking experiments also confirm that flocking allows pigeons to pool information [17] and achieve more efficient routes [24], as predicted from theoretical studies. However, there are still no empirical data on how flock decisions emerge from birds' momentary responses to each other and to the environment.

In this study, we measure flocking responses from movement data on 80 co-navigating pairs of homing pigeons, recorded with high-resolution GPS (Global Positioning System) loggers. We compare each pair's track to the pigeons' previous solo tracks from the same release point, to determine whose navigational information the pair followed. With the aid of a model informed by the collective motion data, we investigate how leadership emerges from individual differences in flight behaviour, measured during the solo flights. We then compare pigeon collective behaviour to that of other species and to the assumptions of previous modelling studies.

## 2. Material and methods

### 2.1. Data collection

We recorded GPS tracks from 23 homing pigeons bred and housed at Oxford University Field Station, Wytham, Oxford, UK. The pigeons were between 1 and 2 years old and all had the same level of homing experience, having been previously released from sites 6 to 8 km to the north and east of the home loft and from shorter distance sites to familiarize them with the area within 3 km of home. All flights in this study were from a site 10.4 km SSW of the home loft (bearing to loft: 26°). We tracked homing flights using GPS data loggers set to record 5 fixes s^–1^ (QStarz BT-Q1300ST, 15 g), attached to pigeons via Velcro strips glued to trimmed feathers on their backs. Each pigeon made up to two homing flights per day, with at least 2 h to rest between flights.

**Figure 1.**
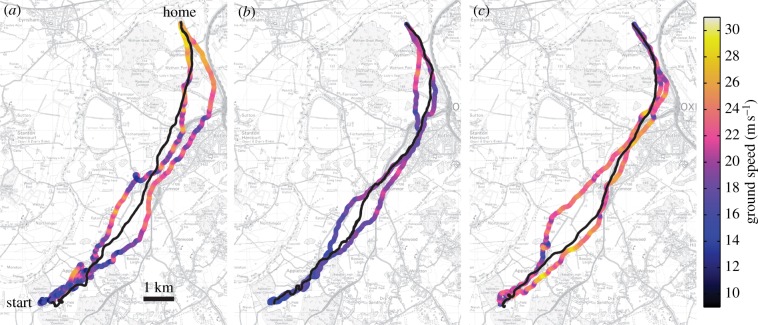
Tracks of pairs of homing pigeons and their previous solo routes. In each panel, black lines show a pair's GPS tracks and shaded lines show the two birds' previous solo tracks, illustrating the conflicts that arise due to differing route preferences. Ground speed is plotted along the solo tracks, smoothed using a 4 s moving average. Ordnance Survey mapping *©* Crown copyright 2012. (Online version in colour.)

First, we released each pigeon singly 21 times to allow it to learn a landmark-based route [23,25]. We recorded GPS tracks of the last five solo flights. To summarize the difference between any two routes, we found the distance to the nearest point on the target track from every point along the focal track, and then took the mean of these distances. For flights 19–21, a bird's distance to its own previous solo route was 160 ± 86 m (mean ± s.d.), compared to 648 ± 459 m to other birds' previous routes, indicating route recapitulation as in previous studies [17,23,25]. We then released pigeons in pairs (figure 1), by placing two birds in a carrying crate and opening a door in the side of the crate. Between paired flights, we released each bird singly again to test whether it retained its established solo route. If the mean nearest-neighbour distance from the previous solo route was more than 275 m (the 90th percentile from flights 19–21), we gave a pigeon additional solo flights until its route achieved this criterion of similarity. No pigeon flew with the same partner more than once. Where possible, we chose pairings with a large distance between solo routes because these cases are more informative about group decision-making. Nonetheless, across the pairs there was a wide range of distances between solo routes, from 69 to 1573 m (measured as mean of point-by-point nearest-neighbour distances). We removed a pigeon from the experiment if its Velcro strip began to detach from the feathers. In total, we recorded 85 paired flights over the course of seven weeks, with 1–12 paired flights per pigeon. We excluded from the analysis five pairs in which the mean distance between birds was more than 200 m. A previous study estimated 200 m as pigeons' perceptual range for flocking [17], so these five pairs that split would have been out of range for a large proportion of the homeward track.

We tested the error in GPS measurements of relative position and direction by fixing two trackers to a pole, 1 m apart, and carrying the pole on a bicycle back and forth along a straight track (approx. 500 m) with a clear view of the sky. We repeated the procedure with the pole either perpendicular or parallel to the direction of travel. At each time step, we calculated the difference (*φ*) between the trackers' measured directions of travel. The spatial error responsible for *φ* (distance travelled × sin*φ*) was normally distributed with a standard deviation of 0.054 m. The total error in measuring relative position was somewhat larger (median 1.69 m, 95th percentile 4.33 m). The total error is consistent over a timescale of minutes and therefore has little effect on direction measurements. This validates the claim in previous studies that velocities measured from GPS have less error than positions [16].

### 2.2. Data analysis

**Figure 2.**
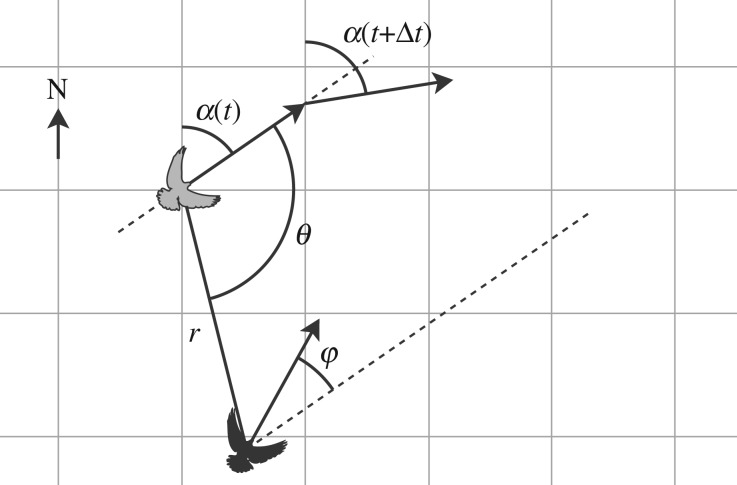
Interaction variables calculated from the pigeon tracks and from the simulation. For the focal individual (grey), we calculated the distance (*r*) and angle (*θ*) to its neighbour (black), as well as the difference in flight direction (*φ*). We estimated the instantaneous direction of travel (*α*(*t*)) from the vector to the next GPS fix. The angles *θ* and *φ* were measured relative to *α*(*t*). (*α*(*t* + *Δt*) − *α*(*t*))/*Δt* gave the focal bird's turn rate. We calculated all variables in the horizontal plane, because the horizontal dimension contains most of the variation between homing routes and is therefore more relevant to route choice.

We converted latitude and longitude to metres using a Universal Transverse Mercator projection and excluded points before takeoff or after landing. At every time step on the paired tracks, we calculated each bird's direction of travel in the horizontal plane (*α*_*i*_(*t*)), which we used to calculate its turning rate, (*α*_*i*_(*t* + *Δt*) − *α*_*i*_(*t*))/*Δt*, and the difference in direction between the two birds (*φ* = *α*_*j*_(*t*) − *α*_*i*_(*t*)). We also calculated the angle (*θ*) and distance (*r*) to the neighbour (figure 2), such that *θ* = 0 when the neighbour was directly in front in the direction of flight, *θ* > 0 when the neighbour was on the right, and *θ* < 0 when the neighbour was on the left. Turn rate and *φ* were also signed negative for anti-clockwise and positive for clockwise. We analysed flocking responses in the combined data from all paired flights.

As a metric of momentary leadership in pairs, we found the time delay (τ*) at which the neighbour's flight direction was maximally correlated to that of the focal bird [16,26]. For this purpose, we calculated the correlation between the focal bird's direction at time *t* and the neighbour's direction at time *t* + *τ*, where −10s < *τ* < 10 s, using the dot product of unit-length velocity vectors (**v**).2.1
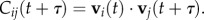


To test how directional correlation delay varies with the neighbour's position, we divided the data into bins based on *r* and *θ*. For each value of *τ*, we averaged *C_ij_*(*t* + *τ*) across all points in the bin, and then found the time delay τ* than maximized directional correlation. Note that *C_ij_*(*t*) = cos*φ*, so τ* is the time delay that minimizes the birds' absolute difference in direction *|*φ*|*. If τ* is positive, the neighbour tends to follow the flight direction adopted by the focal bird, and *vice versa* if τ* is negative.

As a second method of analysing leadership, we analysed the position of the paired routes relative to the preferred solo routes of the two birds. We calculated the distance, *d_i_* from a bird's position during a paired flight to the nearest point on its previous solo route. *Δd_i_* is negative when pigeon *i* approaches its solo route. For a pair of birds flying together (*i* and *j*), *Δd_i_* < *Δd_j_* indicates that their movement is more towards *i*'s route, and in this circumstance we estimate that *i* had more influence over route choice, provided that the solo routes were diverging (*Δd_i_* + *Δd_j_* > 0). We excluded portions of track where the solo routes were converging, for example, when nearing home, which might cause a pair to move towards *j*'s route even when following *i*'s route and regardless of *j*'s influence. If the pair split it is meaningless to classify either as a leader or follower, so we restricted the analysis to times when the pigeons remained within 200 m of each other (perceptual range of flocking estimated by Biro *et al.* [17]).

To investigate individual differences that might predict a bird's position and influence in a pair, we analysed speed and route fidelity in the five solo tracks preceding each paired flight. First, we discarded portions of the solo tracks within 200 m of the release point or the home loft. We calculated instantaneous ground speed along the remaining portion of the track. We quantified route fidelity using the method of Freeman *et al.* [14], which iteratively finds a mean path of 1000 points that minimizes the distance (*d_i_*) to the nearest neighbouring points on the five original GPS tracks. At each point on the mean path, the spatial variance is2.2
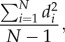
where *N* = 5 in this case. High variance indicates low route fidelity.

Having calculated speed and variance, we compared these solo-flight variables to behaviour in a pair (front–back positioning, influence over route choice). To make the comparison using solo-flight variables from a nearby part of the landscape, we started with a point on the paired track, found the nearest point on each of the pigeon's five preceding solo tracks, and used the mean speed from those five points. Similarly, we used the variance from the nearest point on the mean path. We tested the significance of relationships between solo and pair flight variables using a randomization test, in which we randomly assigned a set of solo tracks to each pair track and then repeated the analysis. The sets of solo tracks each consisted of five consecutive tracks from the same bird, randomly chosen without replacement from the 160 sets preceding paired flights. We obtained a two-tailed *p*-value by comparing the regression slope (*β*) from the real dataset to the distribution of *β* from 10^3^ randomizations.

## 3. Results

### 3.1. Response to partner

**Figure 3.**
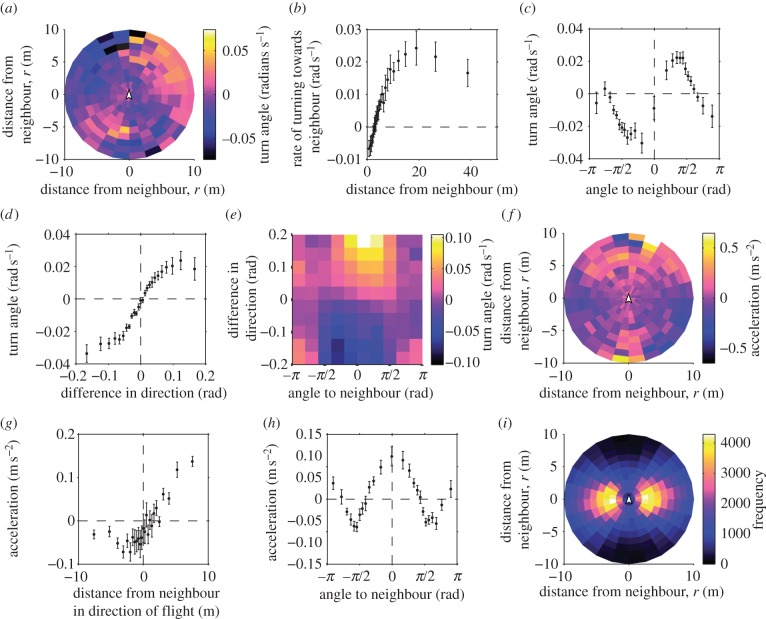
‘Rules’ of flocking interaction. (*a*) Average turning rate of the focal bird versus the relative position of the neighbour. (In these plots, the focal bird is at the origin, facing up.) As shown in *i*, some bins contain very few occurrences of the neighbour and are therefore more likely to assume extreme values. (*b*) Distance *r* to the neighbour versus average rate of turning. (*c*) Angle *θ* to the neighbour versus turning rate. Positive values of *θ* indicate that the neighbour is on the right side; positive values of the turning angle indicate a right turn. Only neighbours at distance *r* > 3 m were considered in the average. (*d*) Alignment response of the focal bird versus difference of orientation to the neighbour *φ*. (*e*) Alignment response of the focal bird versus angle to the neighbour *θ* and difference in orientation *φ*. (*f*) Average change of speed of the focal bird versus the relative position of the neighbour. (*g*) Average change of speed versus the projected front–back distance to the neighbour, *r* cos *θ*. Positive or negative *x*-axis values indicate that the neighbour was, respectively, in front or behind the focal bird. (*h*) Average change of speed versus angle *θ* to the neighbour. (*i*) Frequency of counts of the neighbour being in each particular bin of *r* and *θ*. Note that the bins are not of equal area. Error bars in *b, c, d, g* and *h* show the standard deviations of bin-means from 500 bootstrap replicates, created by randomly sampling the 23 birds, with replacement. (Online version in colour.)

From the GPS tracks of paired flights, we calculated the distance (*r*), angular direction (*θ*) and relative orientation (*φ*) of the partner, and compared these to the focal bird's changes in speed and direction (see Material and methods and figure 2). A pigeon tended to turn towards its neighbour when *r* > 3 m and away from its neighbour when *r* < 3 m (figure 3*a,b*). Turning was strongest when the neighbour was directly left or right of the focal bird (figure 3*c*). A pigeon's turn rate was positively correlated with *φ* (figure 3*d*), indicating alignment with the partner's direction. Rather than being mediated by attraction, this alignment response is in addition to the effect of the neighbour's position (figure 3*e*). Therefore, the highest magnitudes of mean turn rate occurred when *φ* and *θ* had the same sign, in other words when the partner was on the left going left or on the right going right.

Changes in speed also mediate flocking. When the neighbour was more than 2 m in front in the direction of travel, a pigeon tended to speed up, but otherwise tended to slow down (figure 3*f*,*g*). The acceleration, attraction and alignment responses were forward biased, being absent or reversed in most of the range *|*θ*|* > 3*/*5*π* (figure 3*a*,*c*,*e*,*f*,*g*). This forward bias is also prevalent in the fact that the rate of acceleration towards a neighbour in front is higher than the rate of deceleration towards a neighbour behind (figure 3*g*). Speeding attraction peaked when the neighbour was directly in front (*θ* = 0), and turning attraction peaked near *θ* = ± *π*/3 (figure 3*c*,*h*). An immediate result of these various flocking responses was that pairs most frequently flew side by side (i.e. *θ* = ± *π*/2), approximately 3 m apart (figure 3*i*). The pairs were also highly aligned in their flight directions, with median absolute difference in orientation of *|*φ*|* = 0.053 rad. Bootstrap standard errors (figure 3*b*,*c*,*d*,*g*,*h*) indicate that these responses are observed robustly across subjects.

## 3.2. Response to established route

In addition to the flocking interaction, we found that each bird was also attracted towards its preferred route. In some cases, the pigeons flew down the established route of one of the birds, and in other cases they took compromise routes (figure 1). We calculated the percentage of time that the partner and the nearest point on the previous route were on opposite sides of the focal bird, combining data from both birds to give one data point per paired flight. These values had a mean (±s.d.) of 62.9 ± 9.8% and were significantly higher than the 50% occurrence expected by chance (*t*_79_ = 11.8, *p* < 0.01, 99% CI of 60.0–65.8%), which indicates that the pigeons' established routes affected their left–right positioning within the pair. It is further evidence that the birds flying in pairs continued to respond to landmarks along their previous solo routes. The partner and the preferred route are still on the same side a large portion of the time, which is expected given that the pairs did not always fly in between the two preferred routes, and portions of the paired flights have very little conflict of information (figure 1).

We quantified route attraction during solo flights, when there was no confounding influence of conspecifics. The intensity of turning in the direction of the previous solo route was maximized when the bird was approximately 212 m from the nearest point on its previous route (see electronic supplementary material, figure S1). This shape of route response is probably because pigeons tolerate small perturbations within a route corridor but are increasingly motivated to return to the route after larger perturbations [25], counteracted by reduced visibility of landmarks over hundreds of metres. To avoid introducing extra parameters, we made the simplifying assumption that a pigeon is attracted to the nearest point on its preferred route, when in fact a pigeon displaced from its preferred route is more likely attracted to a point downstream, i.e. closer to home [25]. Our approximation realistically captures the behaviour of a pigeon flying roughly parallel to its preferred route, first because attraction either to the nearest point or to a downstream point will generally require turning in the same direction, and second because repeatedly making small turns towards the nearest point will result in the bird re-joining downstream.

### 3.3. Simulation model

To test our understanding of how the birds interact with each other and their environment, we developed an SPP model based on the interaction rules inferred from figure 3. Our model builds on those by, for example, Vicsek *et al.* [2] and Strömbom [6], in which direction changes are mediated by the positions and directions of neighbours. In the model we now propose, we also incorporate the speed changes observed in the pigeons, rather than assuming constant speed. The model allowed us to test the sufficiency of the inferred rules for reproducing patterns of paired movement—both local flocking geometry as well as the decision-making properties of the pair when they had conflicting route information. Furthermore, we could use the model to test the effects of individual differences on the decision outcome, even if these individual characteristics were not directly manipulated in the experiment.

**Figure 4.**
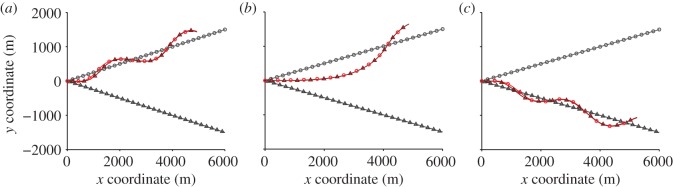
Typical trajectories in separate runs of the simulation of two birds with different preferred speed. Diverging straight lines: preferred routes; curved lines: paired flights. The preferred speed of bird 1 is 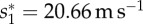, which is equivalent to the average speed in the experiments. The preferred speed of bird 2 is 19.72 m s^−1^ in (*a*), 20.62 m s^−1^ in (*b*) and 21.52 m s^−1^ in (*c*). The faster bird can effectively lead the pair towards its preferred route. (Online version in colour.)

In the model, each bird turns in response to the neighbour's orientation and position and alters its speed to draw level with a neighbour in front or behind. Simulated birds only respond to a neighbour within a visual angle of *|*θ*|* < 3*/*5*π*. We define each simulated bird *i* in terms of its position (*x_i_*(*t*), *y_i_*(*t*)), direction *α*_*i*_(*t*) and speed *s_i_*(*t* + 1). Each bird has its own preferred route, which it will fly towards in the absence of a partner. To simulate a conflict of information, the two preferred routes are assumed to be straight lines that originate at the release point and continually diverge with an angle of 0.245 rad (figure 4). On each time step *Δt* (corresponding to 0.2 s of real time), bird *i* changes its direction according to the sum of four separate response angles, i.e.3.1
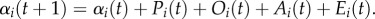

Each of these angles are small and represent the various forces acting on the bird. Below we provide details about the form of each of these components.

The preferred route response is given by3.2
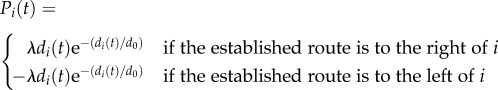
where *d_i_*(*t*) is the distance to the closest point in the preferred route at time *t* and *d*_0_ is the distance from the preferred route at which the attraction reaches its maximum. The parameter *λ* controls the amplitude of turning in the direction of the preferred route. The attraction response function in equation (3.2) had previously been proposed by Biro *et al.* [17], but not fitted to empirical data. We found a good correspondence between this function and the data (see electronic supplementary material, figure S1), with fitted parameters *d*_0_ = 212 m and *λ* = 3.53 × 10*^−^*^4^ rad s^−1^.

Alignment with the neighbour's direction of movement is given by3.3
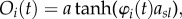
where *φ*_*i*_(*t*) = *α*_*j*_(*t*) − *α*_*i*_(*t*) is the relative orientation of the neighbour at time *t* (figure 2). We use a sigmoidal function (here expressed by the hyperbolic tangent function tanh) to mediate the relation between *φ* and the turning angle, as suggested by the data in figure 3*d*. The slope of the sigmoid is controlled by the parameter *a_sl_* = 14.15 and *a* = 2.79 × 10*^−^*^2^ rad is the asymptotic magnitude of the alignment term. Both the value of *a_sl_* and *a* are obtained by fitting equation (3.3) to the empirical data of pigeons flying in pairs.

Attraction and repulsion to the neighbour are expressed as3.4
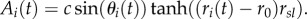


Here, the angle *θ*_*i*_(*t*) = atan2(*x_j_*(*t*) − *x_i_*(*t*), *y_j_*(*t*) − *y_i_*(*t*)) − *α*_*i*_(*t*) gives the direction of the neighbour relative to the position and flight direction of the focal bird (figure 2). Since the focal individual turns away from very close neighbours and towards more distant neighbours (figure 3*b*), we use a sigmoidal function of distance to modulate the transition between repulsion and attraction. Specifically, tanh((*r_i_*(*t*) − *r*_0_)*r_sl_*) is positive when the distance to the neighbour *r_i_*(*t*) is larger than the repulsion radius *r*_0_ = 2.92 (the neighbour is in the attraction zone) and negative when *r_i_*(*t*) < *r*_0_ (repulsion at close range). The parameter *r_sl_* = 0.4 determines how steep the transition is between attraction and repulsion, and *c* = 2.63 × 10*^−^*^2^ rad s^−1^ gives the magnitude of the attraction/repulsion response. Finally, the error term *E_i_*(*t*) is a normally distributed random variable with mean 0 and standard deviation *σ*_1_.

After updating direction according to equation (3.1), the bird will move with speed3.5
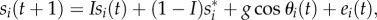
where 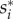 is the preferred speed in the absence of interactions. Bird 1's preferred speed was set to the mean experimental value of 20.62 m s^−1^, and bird 2's preferred speed was randomly chosen from the range (19.62–21.62) m s^–1^. The parameter *I* = 0.9944 is a measure of inertia, *g* = 2.08 × 10*^−^*^2^ m s^−1^ is the strength of response to the position of the neighbour and *e_i_*(*t*) is a Gaussian distributed noise with mean 0 and standard deviation *σ*_2_. This equation expresses the fact that birds speed up when their partner is in front and slow down when the partner is behind but, in the absence of interaction, they progressively revert to adopt their preferred cruise speed s^*^.

The forms of equations (3.2)–(3.5) are summarized in electronic supplementary material, figures S1 and S2. All parameter values were fitted from the empirical data, with the exception of the errors *σ*_1_ = 2.24 × 10*^−^*^2^ rad s^−1^ and *σ*_2_ = 6.7 × 10*^−^*^3^ m s^−1^ and the slope of the transition from repulsion to attraction *r_sl_* (fitted value 0.166), which is related to variability in the repulsion radius. These differences are justified by the observation that a fraction of the measured variability was due to GPS noise and did not reflect real variability in the position and movement of the pigeons.

**Figure 5.**
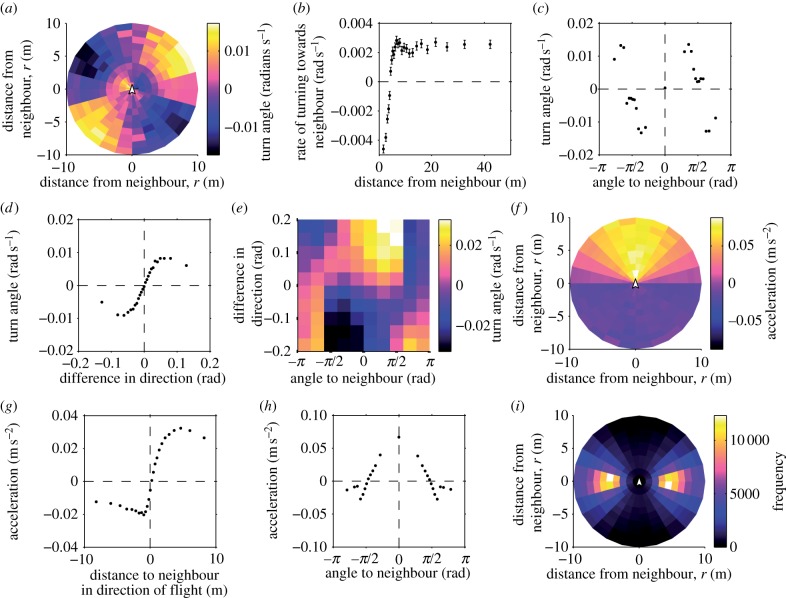
‘Rules’ of flocking interaction apparent in the simulation output. (*a*) Average turning rate of the focal bird versus the relative position of the neighbour. (In these plots, the focal bird is at the origin, facing up.) (*b*) Distance *r* to the neighbour versus turning rate. (*c*) Angle *θ* to the neighbour versus turning rate. Positive values of *θ* indicate that the neighbour is on the right side; positive values of the turning rate indicate a right turn. Only neighbours at distance *r* > 3 m were considered in the average. (*d*) Alignment response of the focal bird versus difference of orientation to the neighbour *φ*. (*e*) Alignment response of the focal bird versus angle to the neighbour *θ* and difference in orientation *φ*. (*f*) Average change of speed of the focal bird versus relative position of the neighbour. (*g*) Average change of speed versus distance to the neighbour projected onto the direction of travel, *r* cos *θ*. Positive or negative *x*-axis values indicate that the neighbour was, respectively, in front or behind the focal bird. (*h*) Average change of speed versus angle *θ* to the neighbour. (*i*) Frequency of counts of the neighbour's occurrence in each particular bin of *r* and *θ*. Note that the bins are not of equal area. Error bars in *b, c, d, g* and *h* (often smaller than symbol size) show standard errors based on the number of points in each bin. (Online version in colour.)

These simple rules reproduce qualitatively many of the observed features of interactions between real birds. In the simulations, as in the data, birds typically flew side by side (figure 5*i*). Some less intuitive aspects of the empirically observed interactions also appeared in the simulation output. In both the simulations and the empirical data, the focal bird turned away from its neighbour when the neighbour entered the blind angle (figures 5*a,c,e* and 3*a,c,e*). The simulation demonstrates that this behaviour can arise without any explicit avoidance response to a neighbour behind, and instead it is due to the higher relative influence of the preferred route once the neighbour enters the blind angle. In the empirical data, the focal bird also presented an acceleration response when the neighbour was directly behind (figure 3*f,h*), whereas there was no such acceleration in the simulation (figure 5*f,h*). This acceleration response might arise if real birds accelerate in response to the preferred route, something we did not implement in the simulation.

The other plots in figure 5 are qualitatively similar to the corresponding plots in figure 3. Most differences between the two figures stem from the fact that in the simulation the birds are always in a conflicting situation. For this reason, simulated birds are observed to turn away from neighbours positioned behind them with greater intensity than real birds. This higher level of conflict in the previous routes also decreases the total signal in figure 5*b*. Furthermore, the intensity of the responses observed from the simulation did not match the data exactly (e.g. figure 5*a,f* versus figure 3*a,f*). Response intensity in the simulations could be manipulated by increasing or decreasing the noise parameter, but the sign of the response typically remained stable. For readers interested in testing combinations of parameters different from those reported in the figures, we make available a commented version of the Matlab simulation code as electronic supplementary material.

### 3.4. Leadership

**Figure 6.**
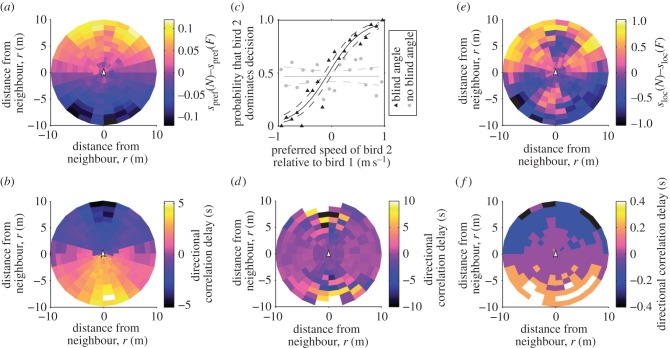
Properties of pigeon movement visualized from the simulation (*a–d*) and the data (*e–f*). (*a*) Difference between the preferred speed of a simulated bird and the preferred speed of the neighbour versus the relative position of the neighbour, for simulations with a blind angle. Birds with faster preferred speed tend to position in front during the simulated paired flights. (*b*) Directional correlation delay versus neighbour's relative position, for simulations with a blind angle. The bird in front usually changes direction first and the bird behind follows. (*c*) Route choice in simulations versus the difference in preferred speed. A bird ‘dominated’ the route choice if the pair ended the simulation closer to that bird's preferred route. Symbols show proportion of simulations that bird 2 dominated, for each of 20 equally spaced bins. Fit lines show logistic regression (±95% CI) for simulations with a blind angle (black line, slope ± s.e. 3.21 ± 0.30, *N* = 401, *p* < 0.001) versus no blind angle (grey line, slope ± s.e. 0.0062 ± 0.17, *N* = 401, *p* = 0.97). The faster bird is significantly more likely to dominate the decision, but only if there is a blind angle. (*d*) Directional correlation delay versus the neighbour's relative position, for simulations without a blind angle. Leadership does not depend on position. (*e*) Difference between the ‘local solo speed’ of the focal bird (the average speed during nearby points on solo tracks, in m s^–1^) and that of the neighbour, as a function of the relative position of the neighbour. Faster birds tended to position in front during the paired flights (slope from linear regression of difference in solo speed versus *r* cos *θ*: real *β* =−0.025, mean ± s.d. of randomized *β* =−5.4 × 10*^−^*^4^ ± 8.8 × 10*^−^*^3^, *p* = 0.004). (*f*) Directional correlation delay times from empirical data versus the neighbour's relative position. In *b*, *d* and *f*, negative delay indicates that the focal bird adopts a particular direction after its partner, whereas positive delay indicates that the focal bird adopts a direction before its partner. (Online version in colour.)

In the model, we assume that each bird has its own preferred speed of flight, with one bird slightly faster than the other. The two simulated birds converged on a common speed, but the bird with faster preferred speed was more frequently positioned in front (figure 6*a*). The model further predicts that, because alignment and attraction are forward biased (i.e. limited visual angle), the bird in front will have a disproportionate influence over directional decisions by the pair. We quantified leader-follower asymmetry using two metrics that reflect different scales of decision-making. On a small spatial and temporal scale, we quantified influence over momentary changes of direction using directional correlation delay [16]. In the simulations, the bird in front tended to initiate turns and was followed by its neighbour behind (figure 6*b*). On a more global scale, we tested which bird dominated the pair's choice of route. The simulated bird with the faster preferred speed consistently led the slower bird towards its preferred route, provided that the simulation included a blind angle (figures 4 and 6*c*). In simulations without a blind angle, getting in front did not give a bird more influence, either measured using directional correlation delay (figure 6*d*) or from the global route decision (figure 6*c*).

These predictions are confirmed in the data. Because solo speed varies along the route (figure 1), we compared pair behaviour to nearby portions of solo track (see Material and methods). Out of the two birds in a pair, the bird with a faster local solo speed tended to be in front during paired flights (figure 6*e*, *p* = 0.004 from randomization test). The bird in front also had a positive directional correlation delay time, indicating that it tended to lead momentary changes in direction (figure 6*f*). To establish which bird had more influence over route choice, we determined which solo route the pair moved towards, and which they moved away from, during the portions of track where the solo routes diverged (see Material and methods). The bird in front was significantly more likely to be successful in leading the pair towards its preferred route (slope from logistic regression of *r*cos*θ* versus route leadership, *β* =− 0.016, mean ± s.d. of randomized *β* = −3.2 × 10*^−^*^4^ ± 3.2 × 10*^−^*^3^, *p* < 0.001). Local solo speed was positively correlated with route leadership, but not significantly (slope from logistic regression of difference in solo speed versus leadership, *β* = 0.039, mean ± s.d. of randomized *β* = 0.052 ± 0.014, *p* = 0.34). There was no significant effect of the variance among the five preceding solo routes (slope from logistic regression of variance*^−^*^2^ versus leadership: real *β* =−8.9 × 10*^−^*^5^, mean ± s.d. of randomized *β* = 1.3 × 10*^−^*^4^ ± 2.9 × 10*^−^*^4^, *p* = 0.46).

## 4. Discussion

With high resolution GPS tracking of co-navigating homing pigeons, we have characterized the momentary responses that form the basis of flocking behaviour. Our simulations based on these ‘rules’ of interaction are able to reproduce several key phenomena in the empirical data. Furthermore, we used a combination of data and simulation to investigate how individual differences affect the outcome of group decisions when there is a conflict of information between birds.

The attraction, alignment and avoidance responses we observed support the assumptions of many SPP models [2,3,7]. The attraction response was sustained over a distance of at least 40 m (figure 3*b*), which suggests that attraction does not have a short-range metric limit that would restrict interactions within large pigeon flocks. Instead, there might be a topological limit to interactions, as data on starling flocks suggest [27], which could be investigated in larger pigeon flocks. At very long range, there is likely to be a metric limit at which pigeons cannot easily see each other and therefore stop interacting (previously estimated at 200 m [17]). In addition, our data indicate an effective ‘blind angle’ for neighbours located behind. The blind angle should not necessarily be understood as a region in which visual or sensory perception is impossible, but simply that pigeons do not normally respond to conspecifics within this region.

In contrast to pairs of shoaling fish [26,28], pigeons displayed strong and explicit alignment behaviour. In other respects, the interaction rules of pigeons are broadly similar to those found in fish shoals as well as surface-swimming ducks [29]. Our observation of a distance-dependent transition from avoidance to attraction is similar to findings of Kattas *et al.* [30], who also parametrized a model using data on pigeon flocks. However, Kattas *et al.* estimated the transition at a much larger radius (approx. 20 m) because they fit a response to the average position of multiple neighbours, regardless of distance, in flights when some pigeons had separated hundreds of metres from the rest of the flock. The contrasting results demonstrate the difficulty of inferring pairwise behaviour from data on larger groups, because collective behaviour underdetermines the pairwise interactions, i.e. many different models can produce similar collective behaviour [31].

The pigeons most frequently flew side by side (figure 3*i*). This configuration has also been observed within large flocks of starlings, where each bird's nearest neighbour is most frequently positioned at *θ* =±*π*/2 in the horizontal plane [27]. Pigeons and starlings contrast with several fish species, which tend to have the nearest neighbour in the school directly in front or behind [26,28]. We can explain the pigeons' spatial configuration mechanistically from the interaction rules we observed. Because repulsion is mediated by turning (figure 3*b*) and not by changes in speed (figure 3*g*), flying side by side is the only stable configuration in which neither bird adjusts its position with respect to the other [7]. Fish, on the other hand, tend to avoid each other by changing speed, leading to an oblong school shape with fish travelling one behind the other [26,32]. These differences in how pigeons and fish maintain distance from each other may be due to the biophysics of their locomotion. For a bird in flight, slowing down would reduce lift, so making small turns may be a more aerodynamic and energetically efficient way for a bird to maintain its position relative to its neighbours [7]. Whereas flocking is energetically costly to pigeons [33], there are other bird species that benefit aerodynamically from flocking. It would therefore make an interesting comparison to measure the rules of motion that give rise to aerodynamically efficient, V-shaped flocks, such as those of geese or pelicans [34].

In addition to mechanistic reasons for a side-by-side configuration, there are several possible functional explanations. When flying side by side, neither pigeon is in the blind angle of the other, which will help maintain flock cohesion and the associated anti-predator benefits [8]. A bidirectional transfer of information between pigeons may also have navigational benefits. If birds attend to each other mutually, leadership remains dynamic in that it can shift according to which bird has the best local information. Such information-based leadership has already been demonstrated in SPP models [4] and supported by experiments on homing pigeons [15]. Our study goes further in discovering interaction rules that make information-based leadership more robust. The type of flocking interaction we found will destabilize a front–back configuration, making leadership less sensitive to initial conditions and allowing compromise routes that average individual preferences [17,24]. Our findings suggest a mechanism for the reciprocal relationships that Xu *et al.* [35] found in pigeon flocks, but also demonstrate that compromise and leadership are not necessarily distinct strategies at the individual level. They can both arise from a single set of rules sensitive to the neighbour's position.

The flocking responses we have characterized provide a mechanism by which individual differences affect leadership through changing the spatial configuration of a flock. This is a topic that has previously been explored with SPP models [5], but with little empirical data for comparison. In our study, leadership correlated with having a faster ground speed during solo flights. Our simulation shows that a speed difference alone is sufficient to cause one bird to lead. We found that a bird in front was more likely to have a positive directional correlation delay (figure 6*f*), a trend that was previously measured in larger pigeon flocks [16], and which can now be explained in terms of interaction rules. Based on this remarkably simple mechanism, a gradient of individual differences through the population would be sufficient to produce the hierarchical leadership patterns seen in larger flocks [16]. However, that is not to say that speed is the sole cause of leadership in our study. High ground speed implies low tortuosity. These flight characteristics might correlate with homing motivation or navigational certainty. Previous studies of pigeons have implicated the latter as an important factor in leadership [14,15]. These other factors could either act on leadership directly, in the sense of ‘leading by need’, or they could act via increasing one bird's speed. In either case, the principle is similar: one bird accelerates in a particular direction and ‘pulls’ the other bird with it.

Our results and model provide a data-driven, mechanistic explanation of flocking and group decision-making. Rather than formulating an abstract model with as few parameters as possible, we fit our model to pigeon behaviour. In doing so, we reveal important differences between the rules of motion in pigeon flocks versus fish schools. The flocking responses in pigeons give rise to two opposing tendencies. Turning-based avoidance stabilizes a side-by-side configuration, which is optimal for information pooling and allows dynamic, fluctuating leadership. This is the opposite of what was found in, for example, mosquitofish, where collision avoidance was mediated mainly by speed changes, and the fish preferentially assumed a configuration of one behind the other [26]. In addition to allowing a bidirectional transfer of information, flocking interactions in pigeons create a system that is sensitive to individual differences. If these differences are stable over time then one individual can maintain a forward position and have more influence over the group's direction. We therefore demonstrate how leadership emerges from simple, anonymous differences in the population. The next question in group navigation is to scale up the observations to more than two interacting birds.

## Notes

The protocols in this study were approved by the Ethical Review Committee of Oxford University's Department of Zoology.

## Data accessibility

Simulation code is available as electronic supplementary material. GPS track data are available from the Dryad Digital Repository: doi:10.5061/dryad.3k31s.
